# Librational Dynamics of Spin-Labeled Membranes at Cryogenic Temperatures From Echo-Detected ED-EPR Spectra

**DOI:** 10.3389/fmolb.2022.923794

**Published:** 2022-06-29

**Authors:** Rosa Bartucci, Erika Aloi

**Affiliations:** ^1^ Department of Chemistry and Chemical Technologies, University of Calabria, Rende (CS), Italy; ^2^ Molecular Biophysics Laboratory, Department of Physics, University of Calabria, Rende (CS), Italy

**Keywords:** model membranes, Na, K-ATPase, spin label, electron paramagnetic resonance, electron spin echo, echo-detected ED-spectra, librations

## Abstract

Methods of electron spin echo of pulse electron paramagnetic resonance (EPR) spectroscopy are increasingly employed to investigate biophysical properties of nitroxide-labeled biosystems at cryogenic temperatures. Two-pulse echo-detected ED-spectra have proven to be valuable tools to describe the librational dynamics in the low-temperature phases of both lipids and proteins in membranes. The motional parameter, $\alpha^{2}\tau_{C}$ , given by the product of the mean-square angular amplitude, $\alpha^{2}$ , and the rotational correlation time, $\tau_{C}$ , of the motion, is readily determined from the nitroxide ED-spectra as well as from the *W*-relaxation rate curves. An independent evaluation of $\alpha^{2}$ is obtained from the motionally averaged ^14^N-hyperfine splitting separation in the continuous wave cw-EPR spectra. Finally, the rotational correlation time $\tau_{C}$ can be estimated by combining ED- and cw-EPR data. In this mini-review, results on the librational dynamics in model and natural membranes are illustrated.

## Introduction

Steady-state, continuous wave electron paramagnetic resonance (cw-EPR) spectroscopy of nitroxide(NO)-labels (*S* = 1/2, *I* = 1) holds a prominent place in membrane biophysics (Berliner, 1976; Marsh, 1981; Berliner 1998; Hemminga and Berliner, 2007; Marsh, 2019). The success and relevance of spin-label EPR in biomembrane studies is due to the fact that its timescale is optimally sensitive to the nanoseconds and matches the timescale of various molecular motions occurring in membrane components. 9-GHz (X-band) spin-label cw-EPR has notably contributed to the study of the dynamics of proteins and lipids in membranes as well as in reconstituted lipid–protein complexes and in lipid model systems (Borbat et al., 2001; Marsh, 2008; Klare and Steinhoff, 2009; Guzzi and Bartucci, 2015; Sahu and Lorigan, 2021).

**FIGURE 1:**
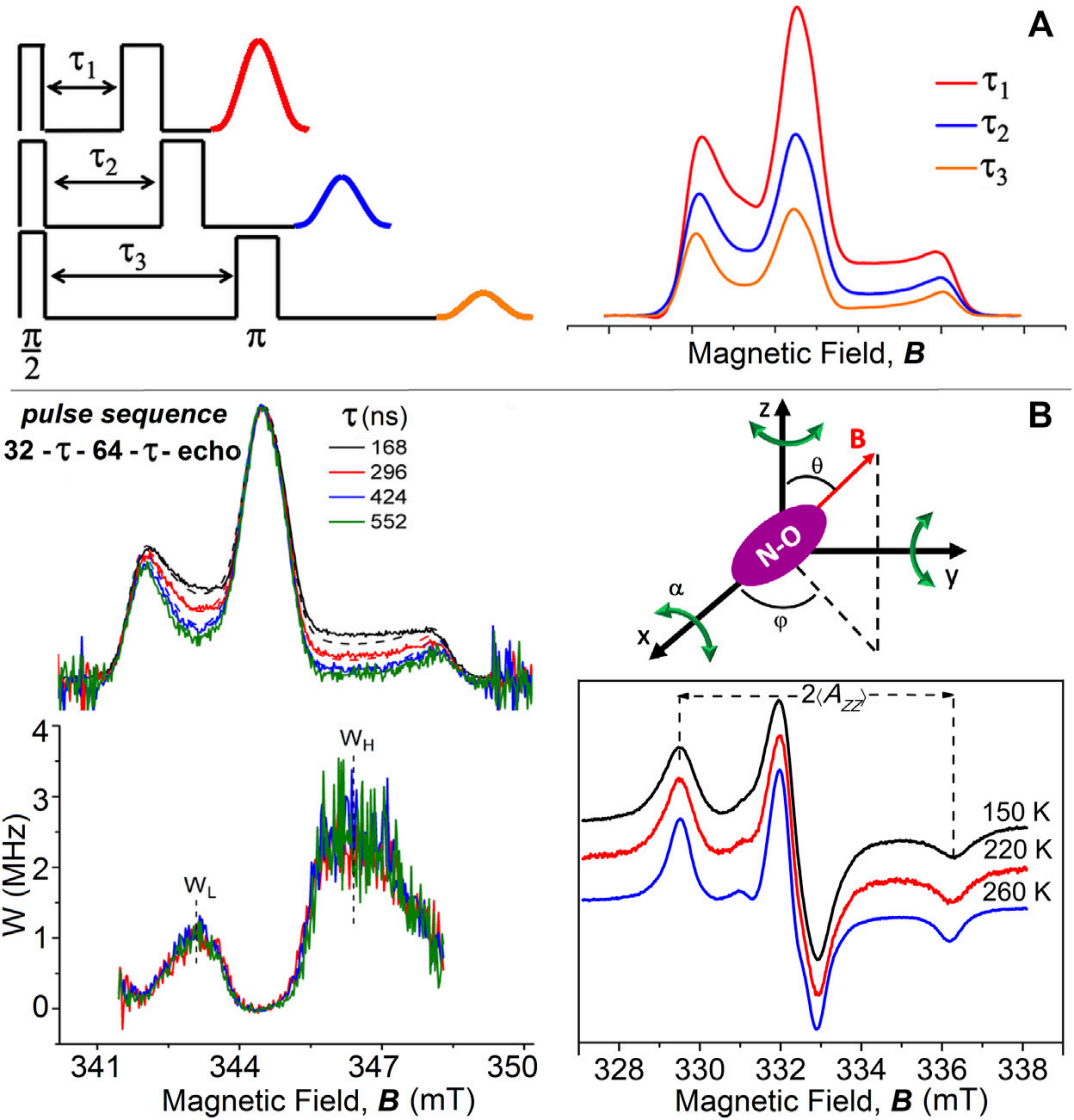
**(A)** Two-pulse primary echo sequences and echo amplitudes decrease with increasing the interpulse delay time, τ; simulated examples of corresponding echo-detected ED-spectra of chain-labeled nitroxide in membranes. **(B)** Two-pulse (π/2-τ-π with microwave pulse widths of 32 and 64 ns) ED-EPR spectra of 5-PCSL in DPPC bilayers at T = 200 K recorded at incremented interpulse spacings τ (from top to bottom). Solid lines are the normalized experimental spectra, and dashed lines are simulations for isotropic librational motion. Underneath are reported the anisotropic part of the relaxation rate, *W*-spectra, obtained according to Eq. 1 from pairs of spectra with interpulse separations of *τ*_1_ = 168 and *τ*_2_ = 296 ns, *τ*_1_ = 168 ns and *τ*_2_ = 424 ns, or *τ*_1_ = 168 ns and *τ*_2_ = 552 ns. Schematic illustration of isotropic librational motion: the nitroxide molecule performs oscillations of small angular amplitude, α, about the three nitroxide axes. cw-EPR spectra of 5-PCSL in DPPC bilayers at 150, 220, and 260 K. ED-, W-, and cw-EPR spectra are taken from Aloi et al. (2017).

Insights into the dynamics of spin-labeled membrane components emerged from the use of electron spin echo (ESE) methods of time-resolved, pulse-EPR spectroscopy (Freed, 2000; Bartucci et al., 2006; Dzuba, 2007). ESE methods are based on the use of resonant microwave power pulse sequences of defined short-time duration, typically 12–64 ns, separated by time intervals in which the microwaves are off, that produce an echo signal at a given delay time (Kevan and Bowman, 1990; Schweiger and Jeschke, 2001). The standard two-pulse sequence, π/2-τ-π-τ-primary echo (Figure 1A), allows experiments on the time domain of the interpulse time spacing τ, determined by the transverse phase memory time *T*_2M_ of the spin-labels, and the two-pulse ESE technique is optimally sensitive to the spin-label dynamics in the nanoseconds timescale. The primary echo, recorded at 2τ from the first pulse, is the result of the refocusing of the spin magnetization after the action of the microwave pulses. The first π/2 pulse flips the magnetization by 90° into the X-Y plane perpendicular to the *Z* direction of the spectrometer magnetic field, B. The spins then dephase during τ, with the time constant T_2M_, until the inverting *p* pulse reverses the magnetization that will refocus after a time τ producing the echo signal. By integrating the echo while sweeping the static magnetic field, an echo-detected ED-EPR absorption spectrum is obtained, the lineshape of which reflects the angular orientation of the spins. For spin relaxation, the echo amplitude decays exponentially when the interpulse separation τ is incremented, and the corresponding collected ED-spectra show variations in the lineshapes (Figure 1A). Such ED-spectra directly reflect the amplitude and the rate of motion of spin-labeled biosystems and contain all the information on their dynamics. Low, cryogenic temperatures are required for ESE-based measurements because spin-labeled T_2M_-relaxation time is generally too fast to produce detectable echoes at an ambient temperature. Thus, ED-EPR spectra offer a convenient route to study the dynamics of spin-labeled biosystems at low temperatures, for samples cooled with liquid nitrogen down to 77 K or with helium below 77 K. Moreover, low-temperature studies are advantageous to reveal dynamical features that occur also at higher physiological temperatures where they cannot be resolved explicitly because they are hidden by large-amplitude motions.

Here, we review results obtained on the low-temperature dynamics of spin-labeled lipid bilayers and natural Na,K-ATPase membranes from two-pulse ED-EPR spectra.

### Two-Pulse ED-EPR Spectra of Nitroxide Labels in Membranes

The pioneering work of Millhauser and Freed, (1984) showed the sensitivity of the two-pulse echo-induced EPR spectrum for each value of interpulse separation time *τ* to variation across the spectrum of the transverse relaxation time. With this ESE technique, the structure and dynamics of cholestane spin-label in oriented lipid multilayers were studied (Kar et al., 1985). Two-pulse ED-spectra have been used by Dzuba et al. (1992), Dzuba (1996), Dzuba (2000), and Kirilina et al. (2001) to investigate the motion of spin-probes in glassy media. The lineshapes, revealing anisotropic phase relaxation, showed a decrease of the amplitudes in the intermediate spectral regions at low and high field with increasing *τ*. The ED-spectra have been simulated by assuming the occurrence of librational motion, that is, an orientational molecular motion consisting of fast, low-amplitude oscillations near an equilibrium position.

An analogous dependence on *τ* has been observed later for the ED-spectra of chain-labeled lipids in model membranes (Bartucci et al., 2003; Erilov et al., 2004a; Erilov et al., 2004b). In these spectra, the regions at intermediate low and high fields, which correspond to the maximum variation of spin orientation with the static magnetic field, relax faster than the others, and the intensities decrease systematically with increasing the interpulse spacing, *τ*. Minor changes are instead observed in the outer peaks, which correspond to stationary turning points (Figure 1B).

The ED-EPR spectra of lipid spin-labels in bilayers are successfully simulated according to the so-called “isotropic” model of librations (Erilov et al., 2004b). The model assumes that librations consist of independent and simultaneous rapid oscillations, each of small angular amplitude *α* and with correlation time τ_*C*_, around each of the three perpendicular *X*-, *Y*-, and *Z*-axes of nitroxide (Figure 1B). For fast motion of small amplitude, that is, *Δω* ^*2*^ *τ* ^*2*^_*c*_ << 1, and for a polar orientation *θ, φ* of the magnetic field, *B*, relative to the nitroxide *X*-, *Y*-, and *Z*-axes, the amplitude of a two-pulse echo decay is approximatively described by $E\left(2\tau ,\theta ,\varphi \right)\approx \exp\left(-2\tau /T_{2M}\right)\approx \exp\left(-2\Delta \omega^{2}\left(\theta ,\varphi \right)\tau_{C}\tau \right)$ , where *Δω* is the shift in resonance frequency that is induced by the motion and *τ*_*C*_ is the rotational correlation time (Dzuba et al., 1992; Dzuba, 1996). This term is explicitly included as a factor in the echo-detected EPR lineshape, *ED*(*2τ, B*), details of which are reported in Erilov et al. (2004b). From spectral simulations, it is possible to extract the motional parameter, $\alpha^{2}\tau_{C}$ , given by the product of the mean-square angular amplitude, $\alpha^{2}$ , and the rotational correlation time, *τ*_*C*_, of the librational motion.

An alternative scheme of analyzing the dependence of the ED-EPR lineshapes on librational dynamics is given by the *W*-relaxation spectra. They are obtained from the experimental ED-spectra recorded at two different values, *τ*_1_ and *τ*_2_, of the interpulse delay by using the relation (Erilov et al., 2004b):

$$W\left(B,\tau_{1},\tau_{2}\right)=\ln\left[\frac{ED\left(2\tau_{1},B\right)}{ED\left(2\tau_{2},B\right)}\right]\cdot \frac{1}{2\left(\tau_{2}-\tau_{1}\right)}.$$ (1)

The *W*-spectra evaluated for different pairs of *τ*-values coincide within the noise level (Figure 1B), showing exponential anisotropic spin relaxation as a function of *τ* (especially on the low-field side), as expected for the isotropic model of librations. The relaxation rate *W*-curves are characterized by the maximum values, *W*_*L*_ and *W*_*H*_, determined in the low- and high-field regions, respectively, of the ED-spectra. The difference in intensity at the two positions arises simply from the different inherent sensitivities of the two spectral regions to spin relaxation.

The relaxation rate *W*_*L*_ or *W*_*H*_ can also be used to characterize the librational dynamics in membranes. Indeed, they are related to the motional parameter $\alpha^{2}\tau_{c}$ *via* the calibration constant, *C*_cal_, established from simulations. For example, *W*_*L*_ or *W*_*H*_ = (*C*_cal_ rad^−2^s^−2^) × $\alpha^{2}\tau_{c}$ (Erilov et al., 2004b).

To fully describe the librational motion of spin-labels in membranes, it is desirable to know the mean-square angular amplitude, $\alpha^{2}$ , and the rotational correlation time, *τ*_*C*_, of the motion. An independent evaluation of $\alpha^{2}\;$ is obtained by acquiring spin-label cw-EPR spectra at the same low temperatures as those of ED-spectra and measuring the motionally averaged ^14^N-hyperfine splittings, $2A_{zz},$ that is, the separation between the two outer spectral peaks (Figure 1B). For small amplitude librations around the *X*-axis, $\alpha^{2}\;$ can be obtained from the relation: $A_{zz}=A_{zz}-\left(A_{zz}-A_{xx}\right)\alpha^{2}$ , where *A*_*xx*_ and *A*_*zz*_ are the principal values of the hyperfine interaction tensor (Van et al., 1974; Dzuba, 2000). *A*_*xx*_ is obtained from the literature (Marsh, 2019), whereas *A*_*zz*_ is derived by linear extrapolation of $2A_{zz}\;$ vs. temperature data to zero temperature. From Figure 1B, it is evident that $2A_{zz}\;$ decreases with the temperature and, according to the aforementioned expression, to this corresponds an increase of $\alpha^{2}\;$ due to librations. Finally, the correlation time *τ*_*C*_ of librations is evaluated from the quotient of the pulsed $\alpha^{2}\tau_{c}\;$ and the continuous wave $\alpha^{2}\;$ data. In this way, combining two-pulse ED-EPR and cw-EPR spectra, the low-temperature librational dynamics has been fully characterized in a number of spin-labeled membranes and proteins (De Simone et al., 2007; Bartucci et al., 2008; Scarpelli et al., 2011; Guzzi et al., 2012).

An alternative approach to analyze ED-spectra is to evaluate the ratio of the echo amplitude at the two field positions with the largest and smallest anisotropies. For molecular librations, the resulting exponential decay rate *W*_anis_ is proportional to $\alpha^{2}\tau_{C}$ (Isaev and Dzuba, 2008; Golysheva et al., 2018; Golysheva and Dzuba, 2020).

### Segmental Chain Librations of Lipids in Model Membranes

**FIGURE 2:**
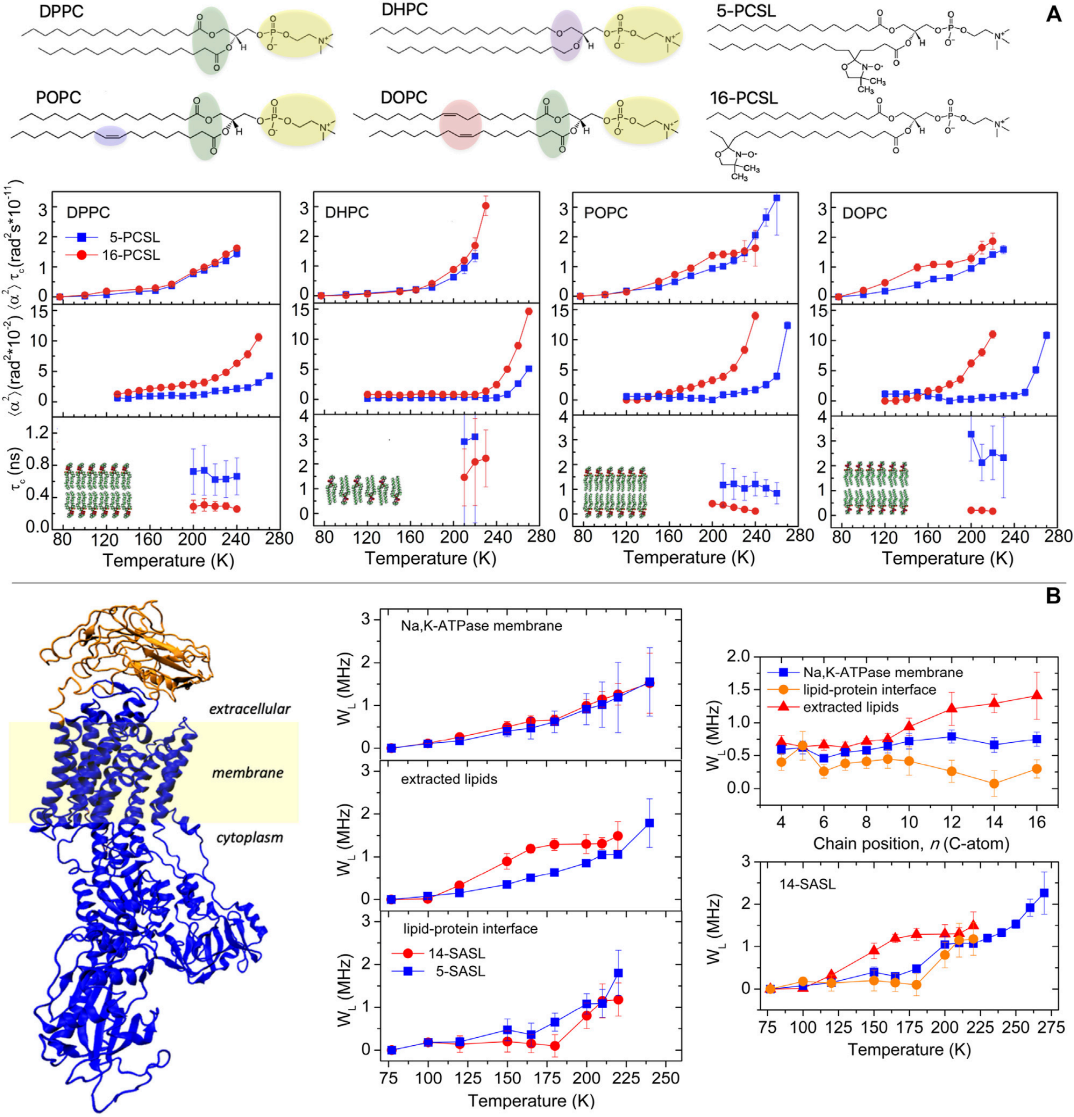
**(A)** Chemical structure of the lipids DPPC, DHPC, POPC, and DOPC and of the chain-labeled phosphatidylcholine spin-label 5-PCSL and 16-PCSL. Characterization of the segmental librational motion in DPPC, DHPC, POPC, and DOPC membranes spin-labeled with 5- and 16-PCSL *via* the temperature dependence of the (i) amplitude-correlation time product, $\alpha^{2}\tau_{C}$ , (ii) mean-square angular amplitude, $\alpha^{2}$ , and (iii) correlation time, $\tau_{C}$ . Error bars for $\alpha^{2}$ are within the symbols. Data for DPPC and DHPC are adapted from Aloi et al. (2017), and those for POPC and DOPC are from Aloi et al. (2019). **(B)** Na,K-ATPase membrane: crystal structure of the enzyme (PDB ID 4RES (Laursen et al., 2015)) and schematic bilayer region. Temperature dependence of the relaxation rate *W*_L_ for 5- and 14-SASL in the Na,K-ATPase membrane, in bilayers of extracted lipids and at the lipid–protein interface. Chain positional profile, that is, *W*_L_ vs. *n*, at T = 180 K of *n*-SASL in Na,K-ATPase membranes, in bilayers of the extracted lipids and at the lipid–protein interface. Temperature dependence of *W*_L_ for 14-SASL in bilayers of extracted lipids and at the lipid–protein interface and for 5-MSL in the Na,K-ATPase protein. Data are adapted from Guzzi et al. (2015).

In this section, we present results on the segmental librations of chain-labeled lipids in the low-temperature phases of model membranes. Bilayers composed of the most prevalent types of lipids present in the cell membrane of the three domains of life, that is, *Eukarya*, *Bacteria*, and *Archaea*, are considered (van Meer et al., 2008; Lombard et al., 2012). They include bilayers of diacylglycerophosphocholine and dialkylglycerophosphocholine lipids which consist of a phosphocholine (PC) polar head group and an apolar region formed by two fatty acid chains covalently bound to a glycerol moiety through ester or ether linkages, respectively (Figure 2A). For the ester-linked diacyl-PC bilayer forming lipids, we used dipalmitoylphosphatidylcholine (DPPC) and the unsaturated palmitoyloleoylphosphatidylcholine (POPC) and dioleoylphosphatidylcholine (DOPC) lipids. For ether-linked lipids, we used dihexadecyl phosphocholine (DHPC), which is analogous to DPPC.

From a biophysical standpoint, the single species lipid membranes show different properties and thermotropic phase behavior (Marsh, 2012). Notably, DPPC and DHPC form bilayers with gel to fluid main phase transition temperature T_m_ ca. 315 K but DHPC spontaneously forms lamellae gel phase with interdigitated chains, whereas DPPC forms noninterdigitated gel phase bilayers. POPC and DOPC, for the presence of cis-bonds in the lipid chain, form low-T_m_ bilayers, T_m_ being ca. 271 K for POPC and ca. 253 K for DOPC. For EPR measurements, the bilayers were spin-labeled with phosphatidylcholine lipids bearing the nitroxide group either at the 5th or at the 16th carbon atom positions of the *sn*-2 chain, namely, 5- and 16-PCSL, to probe, respectively, the first acyl chain segments and the terminal chain region of the hydrocarbon zone of the bilayers (Figure 2A). Lipids and spin-labeled lipids were purchased from Avanti Polar Lipids (Birmingham, AL).

Fast (τ_*C*_ from subnanoseconds to nanoseconds) librations of small amplitude (*α* < 20°) have been detected in DPPC, DHPC, POPC, and DOPC membranes in the low-temperature range of 77–270 K. However, the distinctive features of the lipid acyl chains and the different molecular chain packing between the membranes affect the characteristics of the librational motion.

A temperature-dependent increase of the motional parameter $\alpha^{2}\tau_{C}\;$ is seen in any lipid matrix, indicating that the segmental chain librations intensify with the temperature. In DPPC and DHPC assemblies, the librational oscillations acquire an appreciable intensity from 190 K onward, much more rapidly for interdigitated DHPC lamellae, especially for 16-PCSL. In unsaturated POPC and DOPC bilayers, the librational motion 1) is activated from the lowest temperatures; 2) is more intense in DOPC than in POPC bilayers; 3) in DOPC bilayers, it is more intense at the chain termini in the middle of the bilayers (probed by 16-PCSL) than at the first acyl chain segments close to the polar/apolar interfaces (probed by 5-PCSL) at any temperature (Figure 2A).

The linear and fully saturated acyl chains in DPPC and the interdigitated chains in DHPC impart a well compact and regular packing density to the lipid lamellae in the frozen state which restricts the librational dynamics, at least in the low-temperature regime. In contrast, the presence of double bonds in the hydrocarbon chain of the unsaturated lipids confers a loosened packing density to the bilayers which favors the segmental librations. In agreement with the results in Figure 2A, data on relaxation rates of stearic acid doxyl-labeled along the chain indicated more freedom of segmental chain librations in unsaturated POPC and DOPC bilayers compared to saturated DPPC bilayers (Surovtsev et al., 2012; Golysheva et al., 2018; Golysheva and Dzuba, 2020).

As seen for $\alpha^{2}\tau_{C}$ , the mean-square angular amplitude also increases with temperature in all model membranes (Figure 2A). In frozen bilayers of DPPC, POPC, and DOPC with noninterdigitated chains, $\alpha^{2}\;$ depends on the label position, *n*, along the lipid chain: the amplitude becomes larger on moving from the first acyl chain segments (probed by 5-PCSL) toward the chain termini at the bilayer midplane (probed by 16-PCSL). These results are expected for noninterdigitated lipid bilayers and are in agreement with pulsed EPR results in mixtures of DPPC and equimolar amount of cholesterol and in model membranes composed of lipids extracted from natural membranes (Bartucci et al., 2003; Erilov et al., 2004b; Isaev and Dzuba, 2008; Guzzi et al., 2015). The root-mean-square angular amplitudes in unsaturated bilayers are among the highest obtained. Recently, it has been evidenced by pulse-EPR that the high mobility of unsaturated bilayers is comparable to that of regions of intrinsically disordered proteins (Maslennikova et al., 2021).

In DHPC lamellae with interdigitated chains, the librations are restricted to small angular amplitude at both chain positions of labeling in the low-temperature regime. Only on entering the higher temperature regime, the angular amplitudes increase and are larger at the chain termini than at the beginning of the chain comparable to that in DPPC. Similar results have been obtained in lamellae with interdigitated chains formed by mixtures of DPPC and Lyso-palmitoilphosphatidylcholine or induced in DPPC by ethanol (Aloi and Bartucci, 2019). The behavior of the chain-labeled lipids in DHPC is consistent with the interdigitated phase in which the positional isomers at the chain termini are motionally restricted to an extent comparable to those in proximity of the polar/apolar interface (Boggs et al., 1989; Bartucci et al., 1993; Oranges et al., 2018). At highest temperatures, it is likely that 16-PCSL acquires significant freedom of motion relative to 5-PCSL since it is located in the interfacial region where the polar heads are spaced apart by interdigitation.

From Figure 2A, it can be seen that the rotational correlation time lies on the subnanosecond–nanosecond timescale, indicating that fast rapid segmental chain oscillations are detected in the considered model bilayers. On the whole, the differences in the librational dynamics in the various bilayers are attributable mostly to the variations in the angular amplitude rather than in the rotational correlation time. It is interesting to point out that the temperature dependence of $\alpha^{2}$ shows close similarities with that of the mean-square atomic displacement $r^{2}$ measured in neutron studies (Fenimore et al., 2004; Dzuba, 2007; Golysheva et al., 2017; Peters et al., 2017; Golysheva et al., 2018; Aloi and Bartucci, 2022). Both curves show a rapid increase at a temperature in the range of 200 K ascribed to the dynamical transition from harmonic to anharmonic diffusive motion.

### Librations in Na,K-ATPase Membranes

Membranous Na,K-ATPase is a complex transport system. The lipid bilayer sector is spanned by the sodium pump, a large integral protein (Figure 2B) that is responsible for maintenance of the electrochemical gradients of Na^+^ and K^+^ across the membrane in eukaryotes. Specific regions within the Na,K-ATPase membrane, including the protein, the cationic binding site, and the lipid bilayer environment, have been recently studied by cw- and pulse-EPR of spin-labels and spin-labeled lipids (Guzzi et al., 2009; Guzzi et al., 2015; Guo et al., 2018; Aloi et al., 2021).

The hydrophobic bilayer region of the sodium pump membrane has been investigated exploiting the affinity of ionized chain-labeled stearic acids (*n*-SASL) for the membrane (Bartucci et al., 2014; Guzzi et al., 2015). *n*-SASL was either purchased from Avanti Polar Lipids or synthesized as described elsewhere (Marsh and Watts, 1982). The studies in these samples include measurements of both the Na,K-ATPase membranes and the lipid model systems formed with the extracted membrane lipids and determination of the data at the lipid–protein interface as described in Bartucci et al. (2014) and Guzzi et al. (2015).

The temperature-dependent increase of the *W*_L_-relaxation parameter in Na,K-ATPase membranes is rather similar to that at the lipid–protein interface: the mobility is more evident for T > 180 K and independent on the label position (Figure 2B). It differs notably from that in bilayers of extracted lipids, where mobility is evident from a lower temperature (120 K) and more intense at the end of the chain (i.e., data for 14-SASL) than at the top (i.e., data for 5-SASL) (Guzzi et al., 2015). These features have been confirmed by the positional dependence of the transmembrane librational dynamics. Indeed, the profile of *W*_L_ vs. label position is almost flat for lipid chains at the protein interface and in the Na,K-ATPase membrane where *W*_L_ remains at a relatively low level, comparable to that at the top of the chain in the bilayer lipids. In the lipid bilayers, *W*_L_ is larger toward the end of the chain, with a transition in the region of C10-C12.

Insights into the low-temperature dynamics of Na,K-ATPase have been gained from a comparison of the librational fluctuations of the extracted lipids and interfacial lipids with those of the protein alone studied with a maleimide spin-labels (5-MSL) covalently attached to cysteine–SH residues (Guzzi et al., 2009; Guzzi et al., 2015). The temperature dependence of the *W*_L_-rates for interfacial lipids resembles that of protein side-chains, but not that for the bilayer lipids (Figure 2B). Librational motions of lipids at the protein interface are coupled both to those of the protein and to those of the bilayer lipids: protein and membrane lipids communicate *via* the interfacial lipids. It is most likely that these librational oscillations could drive transitions between the different conformational substates in Na,K-ATPase, which are frozen at lower temperatures but contribute to the pathways between the principal enzymatic intermediates at higher temperatures.

## Conclusion

In this mini-review, we have illustrated the potential of ESE spectroscopy for the study of the nanosecond dynamics in bilayers and Na,K-ATPase membranes at cryogenic temperatures *via* two-pulse ED-spectra. Fast, low-amplitude librations that are readily detected and characterized at cryogenic temperatures must be present in the higher temperature phases of biomembranes, in addition to larger-scale rotational motions. The low, cryogenic temperatures contribute to highlight specific structural, dynamic, and kinetics features of biosystems, and spin-label pulse-EPR results deepen the biophysical characterization of membranes that are normally studied at higher temperatures. Therefore, ESE methods are increasingly used for studying complex macromolecular assemblies.
